# Use of small-angle X-ray scattering to resolve intracellular structure changes of *Escherichia coli* cells induced by antibiotic treatment^1^

**DOI:** 10.1107/S1600576716018562

**Published:** 2016-12-01

**Authors:** A. R. von Gundlach, V. M. Garamus, T. M. Willey, J. Ilavsky, K. Hilpert, A. Rosenhahn

**Affiliations:** a Analytical Chemistry – Biointerfaces, Ruhr-University Bochum, Universitätsstrasse 150, 44780 Bochum, Germany; b Helmholtz-Zentrum Geesthacht, Zentrum für Material- und Küstenforschung GmbH, Max-Planck-Strasse 1, 21502 Geesthacht, Germany; c Lawrence Livermore National Laboratory, 7000 East Avenue, Livermore, CA 94550, USA; d X-ray Science Division, Argonne National Laboratory, 9700 South Cass Avenue, Argonne, IL 60439, USA; e Institute of Infection and Immunity, St George’s University of London (SGUL), Cranmer Terrace, London SW17 0RE, UK

**Keywords:** *Escherichia coli* ultrastructure, antibiotics, small-angle X-ray scattering, SAXS, ultra-small-angle X-ray scattering, USAXS, transmission electron microscopy, TEM

## Abstract

The application of small-angle X-ray scattering (SAXS) to whole *Escherichia coli* cells is challenging owing to the variety of internal constituents. To resolve their contributions, the outer shape was captured by ultra-small-angle X-ray scattering and combined with the internal structure resolved by SAXS. Building on these data, a model for the major structural components of *E. coli* was developed. It was possible to deduce information on the occupied volume, occurrence and average size of the most important intracellular constituents: ribosomes, DNA and proteins. *E. coli* was studied after treatment with three different antibiotic agents (chloramphenicol, tetracycline and rifampicin) and the impact on the intracellular constituents was monitored.

## Abstract 2

A combination of small- and ultra-small-angle X-ray scattering enabled the resolution of important intracellular constituents in *Escherichia coli* (ribosomes, DNA and proteins). The impact of treatment with three antibiotic agents was monitored.

## 1. Introduction   

A broad variety of nanoscale imaging techniques have been established to study the intracellular organization of bacteria. Methods include imaging of thin sections with electron microscopy (Matias *et al.*, 2003 ▸), high-resolution fluorescence light microscopy (Bakshi *et al.*, 2012 ▸) and whole cell imaging with X-ray microscopy (Schneider *et al.*, 2010 ▸). For biological samples, such as bacteria, a large number of cells need to be imaged to obtain statistically significant sampling of the population, which is labor intensive and limited in throughput. Scattering techniques have the general advantage of providing data averaged over a large number of samples (millions of entities) with only seconds of exposure time. Sample suspensions can be used without any preprocessing. However, the technique only provides information on the occurrence of specific size ranges instead of real space images. Nonetheless, small-angle X-ray scattering (SAXS) has matured during the past decade and many synchrotron facilities around the globe provide high-throughput operation and automated analysis pipelines (Graewert & Svergun, 2013 ▸). A major application of SAXS is to study the shape (Blanchet & Svergun, 2013 ▸; Wright *et al.*, 2014 ▸) and function (Fang *et al.*, 2013 ▸) of hydrated proteins. It has also been applied to understanding the organization of soft organic matter like hydrated membranes (Mendil-Jakani *et al.*, 2014 ▸), micelles (Filippov *et al.*, 2013 ▸), human bone tissue (Granke *et al.*, 2013 ▸), human breast cancer tissue (Conceição *et al.*, 2014 ▸) and melanosomes (Gorniak *et al.*, 2014 ▸). Complementary to SAXS is small-angle neutron scattering, with which, for example, the dynamics of hemoglobin within a whole red blood cell (Stadler *et al.*, 2011 ▸) have been investigated. Application of SAXS to complex systems such as entire cells requires specialized data analysis and the correlation with other structure-sensitive methods such as microscopy. We showed recently that the morphological fingerprint of bacteria provided by standard SAXS (*q* ≃ 0.01–4 nm^−1^) is a powerful marker for antibiotic modes of action (von Gundlach *et al.*, 2016 ▸). Because of the complexity of the system (whole bacterial cells) and the limited *q* range, a principle component analysis was used to classify the changes in the bacterial ultrastructure recorded with SAXS. The correlation with transmission electron microscopy (TEM) suggested that the distribution of DNA located in the bacterial nucleoid was a major contribution to the changes observed in the SAXS signal.

In the present study we acquired scattering data across a large *q* range (0.002–3.5 nm^−1^) covering the outer dimensions of *Escherichia coli* and developed a model to analyze the obtained scattering curves. The simplified model considers different intracellular objects, on the length scales of ribosomes, DNA and proteins. Structural changes after the addition of antibiotics were determined and analyzed by this new model. We selected inhibitors of the protein synthesis (tetracycline and chloramphenicol) and an inhibitor of the RNA synthesis since they are expected to change the internal composition of a cell. The presented analytical model is another building block to understand the morphological changes happening in *E. coli* cells during antibiotic treatment and will foster the use of SAXS as screening method for novel antibiotic modes of action.

## 2. Materials and methods   

### 2.1. Sample preparation   

*Escherichia coli* samples (K12, wild type, DSM 498, ATCC 23716) from overnight cultures were diluted in Mueller–Hinton broth (1:40) and incubated at 310 K until an optical density (OD_600_) of 0.45 was reached. This culture was in the exponential growth phase and had approximately 10^8^ cells ml^−1^. The antibiotics [chloramphenicol (60 µg ml^-1^), tetracycline (30 µg ml^−1^) and rifampicin (100 µg ml^−1^)] were each added to 1 ml of inoculum and incubated for 4 h at 310 K. After centrifugation, the bacterial pellets were washed with piperazine-*N*,*N*′-bis(2-ethanesulfonic acid) (PIPES) buffer (0.1 *M*, pH 7.0) and fixed in a 2.5% glutaraldehyde solution in PIPES buffer. To remove the fixative, the samples were washed three times in phosphate-buffered saline (PBS) (10 m*M*, pH 7.0) and stored at 277 K. The final sample volume was 100 µl.

### 2.2. Small-angle X-ray scattering   

The SAXS experiments were performed at the P12 BioSAXS beamline at PETRA III (EMBL/DESY) in Hamburg, Germany. The beamline delivers photons with an energy of 12.8 keV to a spot size of 0.2 × 0.1 mm with a flux of 1× 10^13^ Ph s^−1^. The diffraction patterns were collected with a Pilatus 2M detector (Dectris, Switzerland). A sample robot was employed to collect the diffraction patterns. The 20 µl of cell suspension was delivered automatically by sample robot into a glass capillary (293 K) and the illuminated volume contained roughly 10^6^ fixed *E. coli* cells. The cell density was approximately 10^10^ ml^−1^. In order to obtain a homogeneous suspension, the samples were resuspended with a pipet prior to the measurements. Twenty diffraction patterns were collected for every sample, each with an exposure time of 0.05 s. The PBS buffer was measured before and after every measurement, and the average of the two measurements was used as background and subtracted from the sample curve. To avoid radiation damage by subsequent illuminations, curves showing deviations were discarded by the automated data acquisition software (Franke *et al.*, 2012 ▸). The instrument was calibrated using silver behenate and the observed *q* range was 0.01–4 nm^−1^ (Blanton *et al.*, 2000 ▸).

### 2.3. Ultra-small-angle X-ray scattering   

Ultra-small-angle X-ray scattering (USAXS) experiments were performed on the USAXS instrument at beamline 15ID (now located at the 9ID) at the Advanced Photon Source (APS), Argonne National Laboratory, in Argonne, USA. The beam size was 1 × 2 mm with a photon flux of 10^13^ Ph s^−1^ and a photon energy of 17 keV. The Bonse–Hart camera (Ilavsky *et al.*, 2009 ▸) was operated in slit smeared configuration with Si(220) collimator and analyzer crystals; an Si photodiode was used for detection (Ilavsky *et al.*, 2013 ▸). The observed *q* range was 1.6 × 10^−3^–0.12 nm^−1^. The samples were delivered in suspension in PCR tubes with a cell density of approximately 10^10^ ml^−1^. The beam was centered optically on each sample. The USAXS data were processed with the *INDRA* data reduction package (Ilavsky *et al.*, 2009 ▸) for *Igor Pro* (Wavemetrics, Portland, USA).

### 2.4. Data analysis   

Inhomogeneities in the electron density are the origin of the scattering signal *I*(*q*) recorded in SAXS. The scattering vector magnitude *q* is calculated as 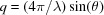, where 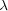 is the X-ray wavelength and 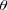 is half of the scattering angle. Inhomogeneities in the electron density are modeled as solid particles with homogeneous density. For multiple (*k*) populations of particles with known shapes the scattering signal *I*(*q*) can be modeled using suitable scattering form factors *F*(*q*, *r*) (Glatter & Kratky, 1982 ▸; Ilavsky & Jemian, 2009 ▸):
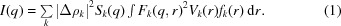
Here 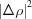 is the scattering contrast, *S*(*q*) accounts for interactions between particles and *V*(*r*) is the volume of a single particle. Polydisperse solutions can be described by the volume size distribution *f*(*r*), which describes the volume occupied by particles of a certain size. All these contributions are functions of the radius of a scatterer *r* and the scattering vector magnitude *q*. The volume size distribution *f*(*r*) is related to the number size distribution *N*(*r*) by
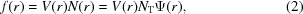
where *N*_T_ is the total number of scatterers and 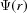 the probability of occurrence of a scatterer at a radius *r*. The data analysis was carried out with the ‘Modelling II’ package of the *IRENA* macros (Ilavsky & Jemian, 2009 ▸) for *Igor Pro*. As first approximation to model the internal cellular particles, the structure factor *S_k_*(*q*) was set to 1, *i.e.* there is no interaction between components.

### 2.5. Merging of datasets   

In the experiments, untreated *E. coli* and *E. coli* treated with chloramphenicol, tetracycline or rifampicin were investigated. The curves for *E. coli* treated with chloramphenicol measured at the BioSAXS and USAXS beamlines had an overlapping *q* interval between 0.005 and 0.01 nm^−1^, which was used for adjusting the relative intensities (Fig. S1). In the other cases, the noise level in the USAXS experiments limited the *q* range. Thus the outer shape of the bacterial cell was modeled as a homogeneous cylinder (Table S1). The model was extrapolated to the BioSAXS data and allowed us to scale the relative intensities (Fig. S2).

### 2.6. Estimation of the applied radiation dose   

The radiation dose was estimated as 1 × 10^5^ Gy at the BioSAXS and 2 × 10^6^ Gy at the USAXS beamline. This is tolerable for the structure for the investigated structure sizes. The calculations followed Howells *et al.* (2009 ▸) and details can be found in the supporting information. The relevant parameters of the BioSAXS beamline were given by Round *et al.* (2015 ▸) and Blanchet *et al.* (2015 ▸) and those of the USAXS beamline by Ilavsky *et al.* (2009 ▸, 2013 ▸).

## 3. Results   

*E. coli* usually has a length of ∼2 µm and a diameter of ∼500 nm. The enclosed intracellular components contain small entities, such as proteins or ribosomes, which are on the scale of a few nanometres. The challenge in investigating bacteria with scattering techniques is that a large *q* range is required if all size ranges from small proteins (a few nanometres) up to the diameter of the bacteria are to be recorded. Most SAXS experiments at both laboratory and synchrotron sources are optimized for proteins, and therefore size ranges of one to one hundred nanometres are accessible. For covering the outer size of *E. coli* one needs to cover size ranges up to 5 µm, which is only possible if scattering at small angles is recorded at ultra-small-angle scattering instruments. In this study we recorded the internal nanoscale information (1–120 nm) at the BioSAXS instrument and combined it with USAXS data (0.1–3 µm) recorded at the USAXS instrument. The difference in photon energy (12.8 keV for BioSAXS and 17 keV for USAXS) leads to a small difference in scattering contrast 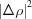 of a whole *E. coli* cell (0.64963 × 10^20^ cm^4^ at 12.8 keV and 0.64973 × 10^20^ cm^4^ at 17 keV) and was neglected for modeling.

**Figure 1:**
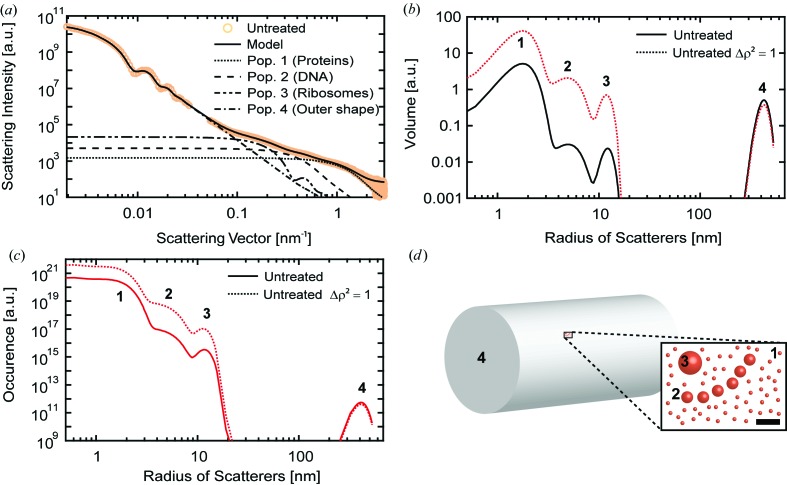
Simplified model for the merged scattering curves of untreated *E. coli* cells. The scattering curves were measured at the P12 BioSAXS (12.8 keV, PETRA III, Hamburg, Germany) and 15ID USAXS (17 keV, APS, Argonne, USA) beamlines. (*a*) Model of untreated *E. coli* using four populations of scatterers. The sizes of the scatterers match the sizes of major cellular components (proteins, DNA and ribosomes) and the outer shape. The outer shape was approximated as a cylinder with fixed aspect ratio and the internal components as spheres. (*b*) Volume distribution *f*(*r*) of the cellular components as a function of their radius. The solid line shows the volume distributions adjusted for the scattering contrasts (Table 1 ▸). The dotted line assumes |Δρ|^2^ = 1 for all scatterers. (*c*) Number distribution *N*(*r*), showing the occurrence of scatterers as a function of their radius. Again, the solid line is adjusted for the scattering contrasts (Table 1 ▸) and the dotted line assumes |Δρ|^2^ = 1 for all scatterers. (*d*) Illustration of the model, featuring a cylinder representing the outer bacterial shape and the major cellular components as spheres. The scale bar has a length of 20 nm.

The outer shape of untreated *E. coli* was modeled as a homogeneous cylinder with a diameter of 840 ± 80 nm and an aspect ratio of 2 (Fig. S2 and Table S1). The internal components of *E. coli* were modeled as filled spheres, the simplest geometrical shape. We did not want to introduce *a priori* information which could not be validated in the data. During fitting it became clear that, in addition to the outer shape, a minimum of three populations of scatterers needed to be modeled to match the ‘shoulders’ in the experimental SAXS curves of the bacterial cells (Fig. 1 ▸). When representing the internal constituents with two populations, no fit of the curve could be obtained, whereas four populations resulted in an undefined model. The mean radii of the three internal populations matched the sizes of several important intracellular components of *E. coli*. The smallest size had a mean diameter of 3.4 nm. This diameter fits well to the size range of many proteins which have an average diameter of 2–5 nm (Erickson, 2009 ▸; Skovgaard *et al.*, 2001 ▸). The second population had an average diameter of 10 nm. This value is close to the typical diameters of three intertwined DNA fibers complexed with histone-like proteins (10 nm) (Ohniwa *et al.*, 2007 ▸; Kim *et al.*, 2004 ▸). The mean diameter of the third population was determined to be 24 nm, which is close to the typical diameter of ribosomes (diameter in crystal structure 21 nm; Schuwirth *et al.*, 2005 ▸). These three normally distributed structural populations are enclosed in the cell wall of the bacterium, modeled as a cylindrical shape with an aspect ratio of 2 and a diameter of 840 nm.

To account for differences in the electron density of internal components and the whole bacterial cell, the respective scattering contrasts were approximated. The scattering contrasts of proteins, DNA and ribosomes were calculated from literature data on the elemental composition and reported densities (Table 1 ▸). For the whole bacterial cell, the average density was calculated from the dry mass composition of *E. coli* (Duboc *et al.*, 1999 ▸) and its water content (Neidhardt & Curtiss, 1996 ▸). This leads to a more accurate description of the internal components and the relative volume they occupy. For comparison, a model with all scattering contrasts |Δρ|^2^ = 1 was calculated, and this is depicted as the dotted lines in Figs. 1 ▸(*b*) and 1 ▸(*c*).

The model best matching the experimental data including approximated scattering contrasts provides a distribution of volumes (Fig. 1 ▸ *b*) and their occurrence (Fig. 1 ▸ *c*). These values were normalized to yield information per single *E. coli* cell. Therefore, the volume/occurrence of internal constituents (populations 1–3 in Fig. 1 ▸) was normalized to the volume/occurrence of the outer cell (population 4 in Fig. 1 ▸). The given number and percentages are always for the average size of a population.

When comparing the absolute values of the volume or the occurrence of internal constituents with literature data, one has to bear in mind that the model is simplified and ignores interaction of particles within the cytoplasm. For DNA–histone complexes and ribosomes, the number and volume estimations are within the same order of magnitude as literature values. The number of ribosomes is 6000 per cell and thus a factor of three off from the literature value of 18 600 per cell (Neidhardt & Curtiss, 1996 ▸). Similarly, the volume fraction is underestimated by a factor of two, being 5% in the model and 10% in literature estimates (Neidhardt, 2008 ▸). The volume content of DNA per cell is 7% and is thus a factor of three off from volume estimations of the bacterial nucleoid of 20% obtained by fluorescence microscopy (Birge, 2006 ▸). The number of proteins is overestimated in our data analysis, being 4 × 10^8^ proteins (average diameter of 3.5 nm) per cell, compared to calculations based on the protein content of the dry mass, yielding 2.5 × 10^6^ proteins per *E. coli* cell (Neidhardt & Curtiss, 1996 ▸). This population includes all cellular structures within a size range of ∼1.5–4 nm in diameter, which are predominantly proteins but also include other cellular constituents such as cell wall components, mRNA and extracellular proteins which may be attached during the fixation step. However, the model values (cell in solution) deviate from the literature values (dried protein mass). The most probable reason is that the model neglects interactions between proteins in the cytosol. This leads to a systematic error in the estimations of absolute cell content. When analyzing changes to the intracellular structures, however, the trends can still be interpreted.

### 3.1. Antibiotic treatment   

**Figure 2:**
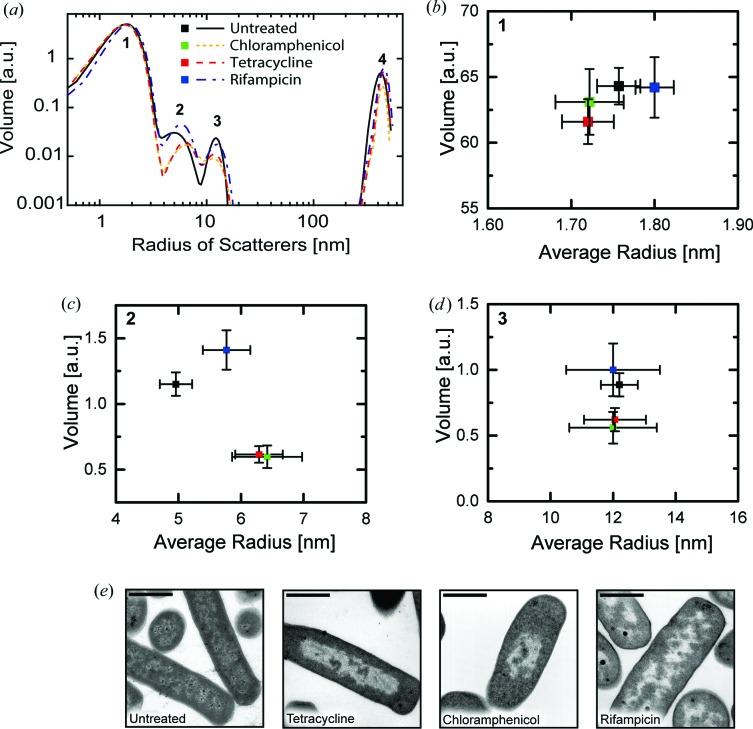
Cellular composition of *E. coli* cells after antibiotic treatment determined by SAXS. (*a*) Volume distribution of the cellular components before and after antibiotic treatment as a function of scatterer’s radius. The total volume and the average radius of each scattering population were extracted from this distribution. (*b*) Average radius and volume of population 1, corresponding to the size range of proteins. (*c*) Average radius and volume of population 2, corresponding to three aggregated DNA fibers covered with histone-like proteins. (*d*) Average radius and volume of population 3, corresponding to ribosomes. The displayed errors bars in (*b*)–(*d*) denote the standard deviation of the model from the experimental data calculated with the uncertainty module of the *IRENA* toolbox. (*e*) Transmission electron micrographs of *E. coli* after antibiotic treatment. The scale bar has a length of 1 µm.

In many cases the bacterial ultrastructure is changed after treatment with antibiotics. We explored how the size and occurrence of the cellular components identified above were affected by incubation with three clinically relevant antibiotics: chloramphenicol, tetracycline and rifampicin. Chloramphenicol is a protein synthesis inhibitor which prevents the formation of new peptide bonds and associates with the 50S ribosomal subunit (Wilson, 2009 ▸). Tetracycline is a protein synthesis inhibitor which binds to the 30S subunit of the ribosome and prevents the binding of a new tRNA molecule by steric interaction (Wilson, 2009 ▸). Rifampicin is an RNA synthesis inhibitor which associates with the bacterial RNA polymerase and blocks the path of the elongating RNA chain by steric interaction (Campbell *et al.*, 2001 ▸). After treatment with the different antibiotics at 10× the minimal inhibitory concentration, bacterial suspensions were investigated by SAXS and USAXS and the obtained scattering data were analyzed in the same way as discussed above. Fig. 2 ▸(*a*) shows the impact of antibiotics on the average diameter of intracellular components and the volume occupied by them.

Treatment with tetracycline had no influence on the mean diameter of the proteins. Also the cellular volume occupied by proteins remained constant. After treatment the volume occupied by DNA was reduced by 50%. At the same time, the average radius of the three aggregated DNA fibers complexed with histone-like proteins was increased by 30%. The impact on the ribosomes was smaller: here the occupied volume was found to be reduced by 30% while the average radius of a ribosome remained constant.

These changes were similar after chloramphenicol treatment, where the protein radius and volume contribution remained unchanged, but the volume of the DNA was reduced by 50%. The volume of the ribosomes was also reduced by 30%. The strong reduction in the volume of the DNA after tetracycline or chloramphenicol treatment is illustrated by TEM images which reveal the condensation of the DNA in the center of the bacterial cell (Fig. 2 ▸ *e*).

The SAXS signal of *E. coli* treated with rifampicin showed an increase of the average radius of the three aggregated DNA fibers complexed with histone-like proteins by 20%. The volume occupied by the aggregated DNA increased by 20%. At the same time, the volume of the ribosomes remained constant, while the size of an individual ribosome was also retained. The size and volume of the proteins remained unchanged. TEM images of rifampicin-treated *E. coli* feature an expanded nucleoid (DNA) (Fig. 2 ▸ *e*).

## 4. Discussion   

The morphological impact of the antibiotics tetracycline, chloramphenicol and rifampicin on the shape of the bacterial DNA, the so-called nucleoid, is well documented in the literature: chloramphenicol and tetracycline con­dense the nucleoid, whereas rifampicin treatment leads to an expansion of the nucleoid (Chai *et al.*, 2014 ▸). The shape of the nucleoid is the result of competing expanding and compacting forces (Cabrera *et al.*, 2009 ▸). A major expanding force is ‘tran­sertion’, which describes the transcription, translation and insertion of membrane proteins into the cytoplasmic membrane. Since this process occurs in close proximity to the cytoplasmic membrane, it anchors the transcribed bacterial nucleoid onto the cytoplasmic membrane (Woldringh, 2002 ▸). The suggested compacting forces include DNA binding of proteins, DNA supercoiling, macromolecular crowding and entropy-driven depletion attraction. Cabrera *et al.* (2009 ▸) suggest that ongoing transcription is necessary for the chloramphenicol-induced nucleoid com­pac­tion. In their fluorescence microscopy observation, a chloramphenicol treatment condensed the nucleoid and a subsequent rifampicin treatment led to complete expansion of the nucleoid.

The TEM images (Fig. 2 ▸ *e*) support this notion as chloramphenicol and tetracycline treatments led to a condensation of the nucleoid while rifampicin facilitated its expansion. The SAXS measurements show that chloramphenicol and tetracycline treat­ments induced a reduction of the overall volume occupied by DNA as suggested by TEM imagery and fluorescence microscopy (Jin *et al.*, 2013 ▸). The aggregated DNA fibers’ increase in radius may be a consequence of stress response where sigma factors attach to the DNA (Figs. 2 ▸ *a*–2 ▸ *d*).

While the morphological effect on DNA is well studied, the effect on ribosomes has not yet been described. SAXS reveals that the size of an individual ribosome after treatment with chloramphenicol or tetracycline (Fig. 2 ▸) remains constant. The volume occupied by ribosomes is reduced, which suggests that an inhibition of the protein synthesis has a reduction of the total number of ribosomes as a consequence.

Treatment with rifampicin has weaker morphological consequences for the bacterial nucleoid. Here, the inhibition of transcription removes the compacting force and the nucleoid expands (Weng & Xiao, 2014 ▸). TEM images confirm this effect (Fig. 2 ▸ *e*). In SAXS we observed that the volume occupied by DNA increases. Simultaneously, we find that the mean radius of the individual DNA fibers increased. We attribute this to relaxation of the fibers as a consequence of a reduced coiling force. The size and volume of the ribosomes remain unchanged.

Summarizing, this work illustrates that SAXS can be used as a structure-sensitive tool to gain information on the internal organization of *E. coli* cells on the nanoscale. The measurement of very low scattering angles allowed deconvolution of the contributions of bacterial outer shape and internal constituents. Inside the bacterial cell, the volume content of DNA and the number and volume of ribosomes can be deduced using a simple model. There are no indications of the occurrence of additional scattering contributions from larger, aggregated mesostructures, such as the bacterial nucleoid. Despite the good fit of the data we have to point out that the applied model is limited since only isolated particles are considered, neglecting any interactions. In order to perform a more holistic modeling, it would be necessary to combine computational models of the cytoplasm with small-angle X-ray scattering data.

Our approach is interesting for modern FEL sources, as sequential single-pulse imaging of large numbers of bacteria allows a complete morphological population analysis of natural isolates or even community analysis of entire biofilms. As intrinsic electron densities were used as contrasting markers, no stains and, apart from incubation and fixation, no preprocessing was required for the experiment. This reduces the effort for sample preparation to a minimum and maximizes the achievable sample throughput. In SAXS, the fact that information averaged over millions of cells can be obtained in seconds makes the method particularly interesting for incorporation into the developmental and testing pipeline for novel antimicrobial compounds.

## Supplementary Material

Supporting information: merging the datasets and estimating the applied radiation dose. DOI: 10.1107/S1600576716018562/jk5008sup1.pdf

## Figures and Tables

**Table 1:** Approximation of the scattering contrasts at 12.8 keV derived from literature data on elementary composition and density The density and elemental composition of a whole bacterial cell were calculated from the dry mass composition (Duboc *et al.*, 1999 ▸) and the water content (Neidhardt & Curtiss, 1996 ▸). The elementary compositions of DNA and ribosomes were calculated from their structure. All contrasts are given relative to H_2_O. (Density: 1 g ml^−1^; |Δ*ρ*|^2^ to vacuum: 88.73 × 10^20^ cm^−4^.) The calculations were performed using the scattering contrast module of the *IRENA* macros (Ilavsky & Jemian, 2009 ▸) for *Igor Pro*.

Component	Density (g ml^−1^)	Approximated element composition	Scattering contrast |Δρ|^2^ (cm^−4^)
Proteins	1.35 (Fischer *et al.*, 2004 ▸)	NC_5_O_2_H_8_	7.983 × 10^20^
DNA	2 (Feijó Delgado *et al.*, 2013 ▸)	PN_5_O_7_C_10_H_14_	66.82 × 10^20^
Ribosomes	1.62 (Fenwick, 1971 ▸)	PN_5_O_8_C_10_H_14_	30.20 × 10^20^
*E. coli* cell	1.1 (Duboc *et al.*, 1999 ▸)	C_0.09_H_0.61_O_0.27_N_0.019_ (Duboc *et al.*, 1999 ▸; Neidhardt & Curtiss, 1996 ▸)	0.650 × 10^20^
