# Supplementary material for: Use of small-angle X-ray scattering to resolve intracellular structure changes of Escherichia coli cells induced by antibiotic treatment
Source: J Appl Crystallogr. 2016 Dec 1;49(Pt 6):2210–6. doi: 10.1107/S1600576716018562 (PMC5139998; doi:10.1107/S1600576716018562)
Supplement: Supplementary file 1 [file j-49-02210-sup1.pdf]

JOURNAL OF
APPLIED
CRYSTALLOGRAPHY

Volume 50 (2017)

Supporting information for article:

Small angle X-ray scattering resolves intracellular structure changes of *E. coli* cells induced by antibiotic treatment

A. R. von Gundlach, V. M. Garamus, T. M. Willey, J. Ilavsky, K. Hilpert and A. Rosenhahn

Supplementary Information

Merging USAXS and SAXS datasets

Since the differences in photon energies have only very little influence on the scattering contrast their impact was neglected. The dataset of chloramphenicol treated *E. coli* featured an overlapping section with identical slope which allowed to adjust the relative intensities (Fig. S1). The different in photon energies were neglected as the scattering contrast is very similar ($|\Delta\boldsymbol{\rho}|^2$ of a whole *E. coli* cell ($\boldsymbol{\rho}$ = 1.1 g ml^{-1} , $C_{0.09}H_{0.61}O_{0.27}N_{0.019}$) is 0.64963 × 10^{20} cm^4 at 12.8 keV and 0.64973 × 10^{20} cm^4 at 17 keV).

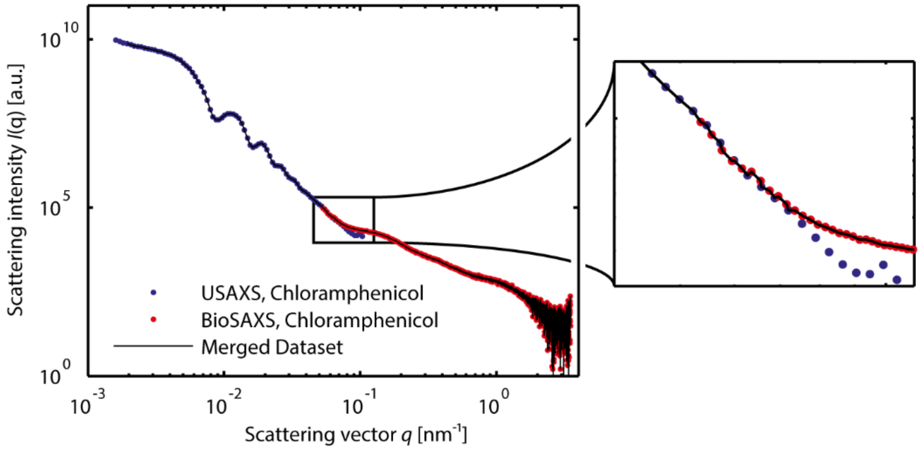

Supplementary figure S1: SAXS curve of chloramphenicol treated, fixed *E. coli* measured at the USAXS (17 keV, APS, Argonne, USA) and the BioSAXS (12.8 keV, PETRA III, Hamburg, Germany). Both datasets show an overlapping section within which both feature the same slope. This overlap allows to scale their relative intensities.

The intensity of the USAXS signal varied with sample density. Thus, the usable q-range which was clearly separated from the noise level was different for each batch of analyzed samples. Only in some cases, the USAXS q-range overlapped with the one of the BioSAXS experiments. The correct relative intensities were found by modelling the scattering of the outer bacterial shape as cylindrical and then extrapolated to the q-range covered in the BioSAXS experiments.

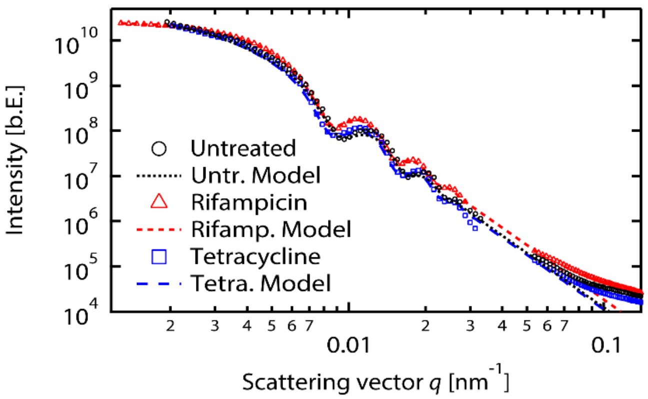

Supplementary figure S2: Merging of the scattering data from USAXS and BioSAXS. The scattering data of the USAXS and BioSAXS did not overlap for the untreated bacterial cells (black) and treatments with rifampicin (red) and tetracycline (blue). Thus the outer shape of the bacterial cell was modeled as a homogeneous cylinder with fixed aspect ratio (Model). The model was extrapolated to the BioSAXS data and allowed to scale the relative intensities. The overlap of the chloramphenicol treated bacterial cells is shown in Figure S1.

A population of normal distributed cyclinders with a constant aspect ratio was found to represent the scattering data of the outer shape in USAXS best. The resulting information is the aspect ratio of the model cylinder, its average radius and standard deviation (width of the distribution). For comparison we quantified the dimensions of bacterial cells in the TEM images, which alwalys display single sections through the bacterial cells. We measured ~ 14 individual cells per treatememt. The comparison indicates, that our USAXS analysis seems to underestimate the aspect ratio.

**Antibiotic**	**Aspect ratio cylinder (USAXS)**	**Average radius *R* [nm] (USAXS)**	**Std. dev. *σ* [nm] (USAXS)**	**Aspect ratio TEM**	**Radius TEM [nm]**
**Untreated**	2	418 ± 10	40 ± 10	3,7 ± 0,8	450 ± 40
**Chloramphenicol**	1	441 ± 10	32 ± 10	2,6 ± 0,6	550 ± 70
**Tetracycline**	2	450 ± 10	34 ± 10	3,2 ± 0,7	550 ±70
**Rifampicin**	1	455 ± 10	40 ± 10	3,2 ± 1,2	600 ± 60

Table S1: Data for the modelling of the outer shape of the bacterial cell as homogeneous cylinders with fixed aspect ratio. The standard deviation around the average radius of the population (normal distribution) indicates the monodispersity.

Estimation of the applied radiation dose

According to Howells et al. (Howells *et al.*, 2009) the radiation dose *D* of a sample with density ρ and absorption coefficient µ can be calculated as:

$$D = \frac{\mu \rho_\lambda E}{\rho}$$

With ρ_λ being the two dimensional photon density with energy E. As density of an E. coli cell was calculated estimated as 1.1 g cm^{-3} with an elemental composition of $C_{0.09}H_{0.61}O_{0.27}N_{0.019}$ (Table 1). An overview of the beamline parameters is given in table S2:

Experiment	Photon flux	Photon energy	Focus size [µm²]	Illumination time	Dose estimate
**BioSAXS**	1×10^{13} Ph s^{-1}	12.8 keV	0.02 mm²	1 s	1×10^{5} Gy
**USAXS**	1×10^{13} Ph s^{-1}	17 keV	0.8 mm²	750 s	2×10^{6} Gy

Table S2: Beamlime parameters of P12 BioSAXS at PETRA III (Hamburg) (Round *et al.*, 2015; Blanchet *et al.*, 2015), and USAXS at APS (Argonne) (Ilavsky *et al.*, 2013, 2009).

The dose was calculated for a cell directly confronting the incident beam for the whole exposure time. The estimated radiation dose was 1 x 10^{5} Gy at BioSAXS. To measure sizes down to 1 nm, a dose of 3×10^{7} Gy would be tolerable (Howells *et al.*, 2009). The radiation dose of the USAXS experiment was estimated as 2×10^{6} Gy. To measure sizes down to 60 nm, a dose of 4×10^{9} Gy can be applied while studying this structure sizes (Howells *et al.*, 2009).
